# Structural Science and Disabilities

**DOI:** 10.1063/4.0001113

**Published:** 2025-10-27

**Authors:** Andrew J Howard

**Affiliations:** Illinois Institute of Technology, Chicago, IL, USA

## Abstract

It is a truism that physical disabilities should not be barriers to success in scientific endeavors, but the reality is that disabilities can be hindrances to success in research. In the ACA we focus on structural science, so I want to draw attention to specific ways that physical disabilities hinder progress in structural science. Blindness, deafness, and limitations in mobility can interfere with effectiveness at the lab bench and even in the computer lab. Less all-encompassing disabilities, such as color-blindness and tremors, can become hindrances—sometimes because of the nature of the tasks, and sometimes because of the conventions by which scientists communicate with one another. I will explore the hindrances, large and small, and the ways that structural scientists can help one another overcome them.

